# Heavy-Tailed Fluctuations in the Spiking Output Intensity of Semiconductor Lasers with Optical Feedback

**DOI:** 10.1371/journal.pone.0150027

**Published:** 2016-02-22

**Authors:** Boon Leong Lan, Cristina Masoller

**Affiliations:** 1 Electrical and Computer Systems Engineering, School of Engineering, Monash University, 47500 Bandar Sunway, Malaysia; 2 Departament de Fisica, Universitat Politecnica de Catalunya, Colom 11, Terrassa 08222, Barcelona, Spain; Technical University of Madrid, SPAIN

## Abstract

Although heavy-tailed fluctuations are ubiquitous in complex systems, a good understanding of the mechanisms that generate them is still lacking. Optical complex systems are ideal candidates for investigating heavy-tailed fluctuations, as they allow recording large datasets under controllable experimental conditions. A dynamical regime that has attracted a lot of attention over the years is the so-called low-frequency fluctuations (LFFs) of semiconductor lasers with optical feedback. In this regime, the laser output intensity is characterized by abrupt and apparently random dropouts. The statistical analysis of the inter-dropout-intervals (IDIs) has provided many useful insights into the underlying dynamics. However, the presence of large temporal fluctuations in the IDI sequence has not yet been investigated. Here, by applying fluctuation analysis we show that the experimental distribution of IDI fluctuations is heavy-tailed, and specifically, is well-modeled by a non-Gaussian stable distribution. We find a good qualitative agreement with simulations of the Lang-Kobayashi model. Moreover, we uncover a transition from a less-heavy-tailed state at low pump current to a more-heavy-tailed state at higher pump current. Our results indicate that fluctuation analysis can be a useful tool for investigating the output signals of complex optical systems; it can be used for detecting underlying regime shifts, for model validation and parameter estimation.

## Introduction

Heavy-tailed fluctuations, where extremely large changes are more probable than the Gaussian prediction, are ubiquitous in finance, and also, in many physical and biological systems. Away from the extreme asymptotic tail regions, such empirical fluctuations are well-modeled by non-Gaussian stable distributions [1]. Examples include the fluctuations of currency-exchange rate and stock price [2, 3], stock market index [4–7], fluctuations of time intervals between water drops from a leaky faucet [8], fluctuations of time intervals between human heart beats [9, 10], fluctuations of animal population [11, 12]. Many other examples can be found in [1].

The class of stable distributions has four parameters [1]: the characteristic exponent, *α* ∈ (0,2], the skewness, *β* ∈ [−1,1], the scale, *γ* ∈ (0, inf), and the location *δ* ∈ (−inf, inf). The class includes the Gaussian (*α* = 2), which has a finite variance; other members (*α* < 2) of the class have infinite variance and heavy tails. The scale parameter, *γ*, provides a measure for the distribution width, and the characteristic exponent, *α*, provides a measure for the tails (smaller *α* implies heavier tails). The location parameter, *δ*, shifts the distribution to the right if it is positive, to the left if it is negative.The sum of independent, identically distributed stable random variables is again stable distributed with the same shape, hence the name ‘stable’.

The non-Gaussian stable model of empirical heavy-tailed fluctuations is useful in a number of ways. The fitted stable parameters can, first of all, be used to quantify the state of a complex system. For example, in the ecological context, the fitted characteristic exponent parameter, *α*, for the fluctuations of animal population can be used to quantify population volatility [12]. In the biomedical context, the fitted scale parameter, *γ*, for the fluctuations of human heartbeat interval can be used to quantify cardiac health state (healthy or diseased) [10]. The fitted parameters can thus be monitored for the purpose of population conservation or health management. In the optical context, the fitted characteristic exponent parameter, *α*, and the scale parameter, *γ*, can be used to characterize coherence enhanced intermittency [13]. Furthermore, the stable fit can also be used to select adequate models and parameters of a system by comparing the fitted stable parameters for the empirical data and the simulated data.

In this paper we analyze the empirical and model data representing the output of an optical system, consisting of a semiconductor laser with optical feedback from an external reflector. The laser operates in the regime of *low-frequency fluctuations* (LFFs), and the output intensity displays a spiking behavior, consisting of abrupt and apparently random dropouts, similar to neuronal spikes. Over the years a great deal of effort has been aimed at understanding the underlying physical mechanisms that trigger the intensity dropouts (in the following referred to as spikes) [14–19]. The laser spiking dynamics has attracted attention due to the complex interplay of intrinsic nonlinearity (light-matter interactions in the laser cavity), various noise sources (with distinct time scales) and high-dimensionality (due to the feedback delay time). Recently, methods using symbolic ordinal analysis have been proposed for identifying signatures of determinism in the sequence of spikes [20–23].

Here we consider experimentally recorded intervals between consecutive optical spikes (referred to as *inter-dropout-intervals* or IDIs, Δ*T*_*i*_ = *t*_*i* + 1_ − *t*_*i*_, with *t*_*i*_ being the time when a spike occurs). While the IDI distribution has received considerable attention [17, 23], the IDI fluctuations have not yet been studied. In fluctuation analysis, the changes between successive values of a quantity are studied [2–13]. The IDI fluctuations are defined, as is typically done in complex systems, as the difference between successive natural-logarithms [5]:

$$\begin{array}{c} \Delta_{n}=\ln\left(\Delta T_{i+1}\right)-\ln\left(\Delta T_{i}\right). \end{array}$$ (1)

For complex systems, studying the changes of a quantity, instead of just the quantity itself, can offer a deeper understanding of the underlying dynamics. Specifically, the analysis of IDI fluctuations provides information about the temporal correlations present between successive IDIs in the time-series, that is, serial correlations, which cannot be inferred from the IDI distribution.

The empirical data studied here, for various values of the laser pump current, is the same as in [23]. We show that the IDI fluctuations are well modeled by non-Gaussian stable distributions. In addition, we show that the Lang and Kobayashi (LK) model [24] generates IDI fluctuations that are also well modeled by non-Gaussian stable distributions, where the fitted stable parameters are not only qualitatively but also, to a certain extent, quantitatively similar to the fitted stable parameters for the empirical fluctuations.

The LK model is a set of two coupled nonlinear delay-differential equations for the complex optical field and the carrier density in the laser cavity. The model includes a time-delayed term in the field equation that represents optical feedback, and a Gaussian white noise term that represents spontaneous emission noise (see Methods for details). While the model has been shown to adequately reproduce many features of the LFF spiking behavior, including the IDI distribution, it is quite surprising that it also reproduces the shape of the distribution of the IDI fluctuations, for the following reason. Because the laser system involves various noise sources which have widely different time scales (optical, spontaneous emission noise is much faster than electrical and thermal noise), it is expected that all these noise sources will play a role in the IDI fluctuations. Thus, taking into account that the LK model employed here is quite simple, it only includes spontaneous emission noise, and neglects relevant effects such as multi-mode emission, thermal effects, spatial inhomogeneities and multiple external reflections, which could also be involved in triggering spikes, the good qualitative experiment-simulation agreement found is quite remarkable.

The rest of this paper is organized as follows: the results are presented in the next section, followed by the discussion and the conclusions. The section Methods includes a description of the empirical data set, the simulations of the LK model, and the diagnostics to assess the stable model of the IDI fluctuations.

## Results

**Fig 1:**
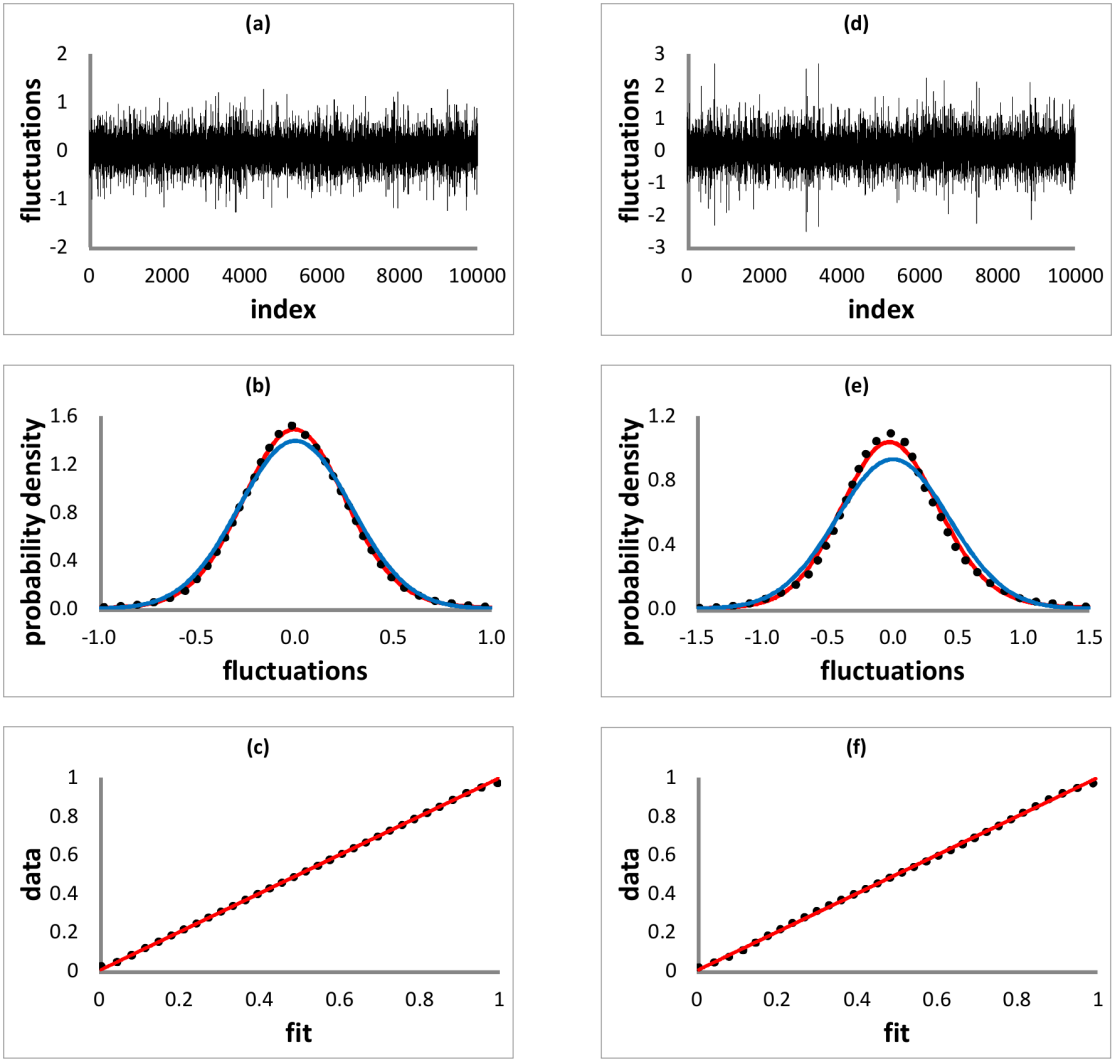
IDI fluctuation, probability density and PP plots. (a)-(c) for the experiment with pump parameter *p* = *I*/*I*_*th*_ = 1.0145 and threshold -1.5, (d)-(e) for the LK model simulation with pump parameter *μ* = 1.020 and threshold -1.0. In the density plots, the red curve is the fitted non-Gaussian stable density, the blue curve is the Gaussian density with the sample mean and variance, and the dotted curve is the smoothed data density. In the PP plots, a 45-degree red line is also drawn for reference.

The experimental and numerical data analyzed is the same as in [23] (see Methods). To assess whether the stable model of the IDI fluctuations is good, two diagnostics, probability density and PP (percent-percent) plots, were used (see Methods). The diagnostics show that the empirical IDI fluctuations are well modeled by non-Gaussian stable distributions. An example from the threshold -1.5 dataset is given in Fig 1(a)–1(c) for experimental pump parameter *p* = *I*/*I*_*th*_ = 1.0145 (that is the pump current normalized to the threshold current), where the fluctuations, defined in Eq (1), are shown in addition to the density and PP plots.

The diagnostics show that the simulated IDI fluctuations are also well modeled by non-Gaussian stable distributions. As an example from the threshold -1.0 dataset, the fluctuation, density and PP plots are given in Fig 1(d)–1(e) for simulation pump parameter *μ* = 1.020 (normalized such that the solitary laser threshold is at *μ* = 1). For both experiment and simulation, Fig 1 shows that there is a good agreement between the fitted non-Gaussian stable density and smoothed data density, and the PP plot is essentially on the 45-degree line. The agreement between the Gaussian density and data density is clearly not as good. Moreover, the D’ Agostino-Pearson statistical normality test shows that all the empirical and simulated fluctuations are not Gaussian distributed (*p* − value <0.0001 in all cases).

The fitted location parameter, *δ*, is essentially zero for both the experimental and simulated IDI fluctuations (see S1 and S2 Tables).

**Fig 2:**
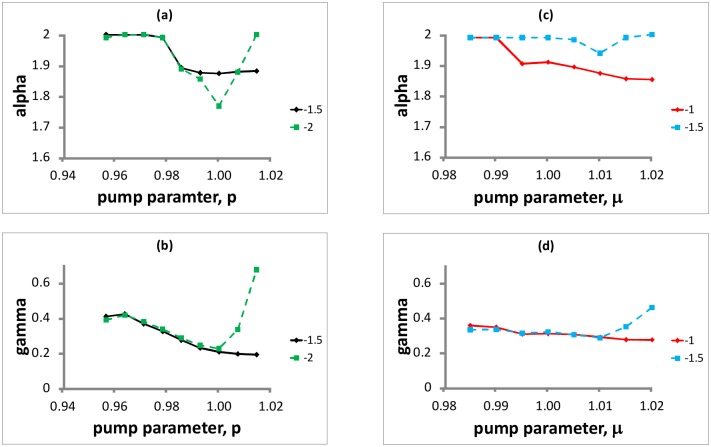
Fitted stable-parameters. *α* and *γ*, versus the pump parameter for two thresholds: (a), (b) experimental data; (c), (d) simulated data. For the experimental data, the maximum values of the 95% confidence interval half-widths are quite small, 0.007 and 0.003 for *α* and *γ*, respectively. For the simulated data, the maximum half-widths are also quite small, 0.01 and 0.004, respectively, and therefore the error bars (parameter +/− confidence interval) are also not plotted because they are all too small to be seen.

For the experimental fluctuations with threshold -1.5, the variation of the fitted characteristic-exponent parameter, *α*, with the pump parameter is displayed in Fig 2(a), where *α* is very close to 2 (*α* = 1.99) at low pump parameters (*p* < 1.0) and decreases to 1.88 at high pump parameters (*p* > 1.0). Since these values of *α* are about 1.9 and above, the variation of the fitted *β* parameter (see S1 and S2 Tables) has negligible impact on the skewness of the stable distribution [1]. The variation of the fitted scale parameter, *γ*, with the pump parameter is shown in Fig 2(b), where *γ* decreases from 0.41 at low pump parameters (*p* < 1.0) to 0.19 at high pump parameters (*p* > 1.0).

When comparing the fitted *α* and *γ* parameters for the simulated IDI fluctuations with two thresholds, -1.0 and -1.5, in Fig 2(c) and 2(d), with the corresponding parameters for the experimental fluctuations with thresholds -1.5 and -2.0, in Fig 2(a) and 2(b), we note that, when the threshold in the simulation is smaller than the one in the experiment (i.e., comparing experimental data with threshold -1.5 with simulated data with threshold -1.0, both represented with solid lines, and experimental data with threshold -2.0 with simulated data with threshold -1.5, both represented with dashed lines), there is a similar trend with increasing pump parameter. In the simulation, as in the experiment, the fitted *α* and *γ* parameters have a similar trend (see Fig 2)—when *α* increases (decreases), *γ* increases (decreases). When *α* and *γ* are larger (smaller), the probability density of the fluctuations is less (more) heavy-tailed but has a larger (smaller) width. The similar trend for *α* and *γ* is simply a consequence of the normalization of the probability density.

Moreover, one can also observe that, for the experimental data set, for threshold -2.0 the fitted *α* and *γ* parameters are close to those for threshold -1.5, except at high pump parameter values. For the simulated data sets, the *γ* parameter is also robust with respect to the threshold, except at high pump parameter values; the *α* parameter is robust with respect to the threshold only at the lowest pump current value. Nevertheless, comparing the left and right columns of Fig 2, one can observe that, for the experiment and simulation, there is also a similar trend in the variation of *α* and *γ* with the threshold.

## Discussion

The results presented in Fig 2 suggest that we can use the fitted stable parameters (*α*, *γ*) for the IDI fluctuations as a novel way to quantitatively define the fluctuation state of the laser. There is one fluctuation state for each pump parameter and the fluctuation state changes continuously and smoothly with the pump parameter (no abrupt change or bifurcation). A fluctuation state with a smaller alpha parameter (thus heavier density tails) implies a large change between successive IDI’s is more probable. A fluctuation state with a smaller gamma parameter corresponds to IDI fluctuations with a smaller spread or ‘amplitude’. For the experimental data set with threshold -1.5, Fig 2(a) and 2(b) show that the laser, which is in the (1.99, 0.41) state at low pump parameters (*p* < 1.0), makes a transition to the (1.88, 0.19) state at pump parameter *p* = 1.01. The IDI fluctuations in the latter state are more heavy-tailed but have a smaller spread. For intermediate pump parameters, the laser fluctuation states are characterized by intermediate values of *α* and *γ*.

In the simulated IDI fluctuations with threshold -1.0 [Fig 2(c) and 2(d)], the laser fluctuation state defined by (*α*, *γ*) is (1.99, 0.35) and (1.85, 0.27), at low (*μ* < 1.0) and high (*μ* > 1.0) pump parameter respectively. Both of these states in the simulation agree quantitatively well with the corresponding states in the experiment, and the transition to the latter state occurs at pump parameter *μ* = 1.015 in the simulation, in good qualitative agreement with the experiment.

Thus, we have shown that the LK model qualitatively reproduces the experimental transition of the laser fluctuation state (*α*, *γ*) from a less-heavy-tailed state at low pump parameter to a more-heavy-tailed state at high pump parameter.

Previous studies have also reported qualitative changes in the laser spiking behavior as the pump current varies: a transition from stochastic spikes at low pump currents to more deterministic spikes at higher currents was reported in [21, 22], and a transition in the probabilities of symbolic patterns was reported in [23, 25]. While there is in principle no clear relationship between an observed more/less heavy-tailed distribution and an underlying more/less deterministic dynamics (because a fully deterministic system can in principle produce both, a no-heavy-tailed and a heavy-tailed distribution), the fact that in the laser system these two transitions are related cannot be excluded, and it will be interesting, for future work, to consider other model parameters and noise levels in order to test if the transition that is detected with symbolic patterns is always accompanied by a transition in the shape of the distribution of IDI fluctuations.

It is unexpected that the LK model reproduces the IDI fluctuations because, for the parameters considered here, without noise the LFF is a transient dynamics. Thus, one could expect that stochastic effects (i.e., the many sources of noise that are present in the experiment –optical, electrical, thermal and mechanical) will ultimately determine spike correlations, which are captured by the fluctuation analysis. However, we demonstrate here that the inclusion of spontaneous emission noise alone (neglecting other noise sources in the experiment) is sufficient to obtain a distribution of IDI fluctuations that is qualitatively (but not quantitatively) similar to the experimental one.

## Conclusions

To summarize, we have shown that the experimental IDI fluctuations of a semiconductor laser with optical feedback in the regime of low frequency fluctuations are well modeled by non-Gaussian stable distributions, which are heavy tailed. In other words, we have shown that the IDI fluctuation distribution is heavy tailed, and thus, a large change between successive IDIs is more probable compared to a Gaussian fluctuation. We have also shown that simulations of the stochastic LK model are in good qualitative agreement with the experimental findings. In addition, we unveiled a transition from a less-heavy-tailed state at low pump current to a more-heavy-tailed state at higher pump current.

Our results demonstrate that fluctuation analysis can be a useful tool for investigating the output signals of complex optical systems, where often only one out of many variables can be experimentally measured (the intensity). For example, fluctuation analysis could be used for testing laser models or estimating their parameters.

## Materials and Methods

### Datasets

The experimental data analyzed is the same as in [25]. Time-series of the laser intensity were recorded for various values of the pump current, which was the experimental control parameter. The times when the optical spikes occur, *t*_*i*_, were defined in terms of an arbitrary threshold: when the output intensity (normalized such that the standard deviation of the intensity time-series is equal to one) decreases below the threshold a spike is recorded.

Simulations of the LK model were also performed, with the model equations and parameters being the same as in [25], and the same procedure was employed to detect the spike times. The model equations are:

$$\begin{array}{c} \frac{dE}{dt}=\frac{1}{2\tau_{p}}\left(1+\alpha \right)\left(G-1\right)E+\eta E\left(t-\tau \right)e^{-i\omega_{0}\tau }+\sqrt{2\beta_{sp}}\xi , \end{array}$$ (2)

$$\begin{array}{c} \frac{dN}{dt}=\frac{1}{\tau_{N}}{\left(\mu -N-G|E\right|}^{2}), \end{array}$$ (3)

*E* is the slowly varying complex electric field amplitude, *N* is the carrier density, *τ*_*p*_ and *τ*_*N*_ are the photon and carrier lifetimes respectively, *α* is the linewidth enhancement factor, *G* is the optical gain, *G* = *N*/(1 + *ϵ*|*E*|^2^) (with *ϵ* being a saturation coefficient), *μ* is the pump current parameter (normalized to unity at the solitary laser threshold -i.e., without optical feedback, the lasing threshold is at *μ* = 1), *η* is the feedback strength, *τ* is the feedback delay time, *ω*_0_ *τ* is the feedback phase, and *β*_*sp*_ is the noise strength, representing spontaneous emission.

The parameters are [25]: *k* = 300 ns^−1^, *γ* = 1 ns^−1^, *ϵ* = 0.01, *τ* = 5 ns, *β*_*sp*_ = 10^−4^ ns^−1^, *η* = 10 ns^−1^, *α* = 4. It is worth noticing that the numerical control parameter, *μ*, is linearly related to the experimental control parameter, *I*/*I*_*th*_: *μ* = 1 + *K*(*I*/*I*_*th*_−1), where *K* is a constant [18].

We also remark that simulations of the LK model give the instantaneous laser intensity, while in the experiments the detection system (photodiode and oscilloscope) has a finite bandwidth of about 1 GHz. To take this into account the simulated intensity time-series was averaged over a moving time-window of 1 ns before detecting the spike times. In the experiments and in the simulations the pump parameter is varied in a range such that the intensity dropouts are individual, well-defined events: if the pump current is too low, the dropouts are too noisy and cannot be distinguished from the background of noisy fluctuations; on the other hand, if the pump current is too high, the dropouts are too fast, they merge and individual events cannot be distinguished [23, 25].

For detecting the spike times, both in the experiments and in the simulations, the intensity time-series were first normalized to zero-mean and unit standard deviation, and then, spikes were detected when the intensity dropped below a certain threshold [25]. To check the robustness of our findings, we performed the analysis with IDIs obtained by detecting the spike times with two thresholds, -1.5 and -2.0, for the experimental data, and -1.0 and -1.5 for the simulated data. The difference in these threshold values is due to the fact that the spikes of the filtered intensity are less pronounced than in the experimental data (this is likely to be due to the time-window average). For a discussion of the influence of the threshold in the detected spike times, see the Supplementary Information of [25].

Regarding the size of the datasets, the number of data points vary with the laser pump current; however, all datasets have above 20,000 data points which are sufficient for robust fitting of a stable distribution to the data.

### Diagnostics for the stable model

There are no closed formulas for the stable densities (except for the special cases of Gaussian, Cauchy and Levy) and thus, the stable densities have to be obtained numerically, by using the characteristic function of the stable distributions, which in the S0 parameterization is:

$$\begin{array}{ccc} \text{if}\;\alpha & \neq & 1,\\ \Phi (u) & = & \exp(-\gamma^{\alpha }{\left|u\right|}^{\alpha }[1\\ & + & i\beta \tan\left(\pi \alpha /2\right)\left(\text{sign}\left(u\right)\right)\left({\left|\gamma u\right|}^{1-\alpha }-1\right)]+i\delta u);\\ \text{if}\;\alpha & = & 1,\\ \Phi (u) & = & \exp(-\gamma |u|[1+i\beta (2/\pi )(\text{sign}(u))\ln(\gamma |u|)]+i\delta u). \end{array}$$

The experimental and simulated IDI fluctuations were fitted with stable distributions using Nolan’s STABLE program [26], which uses the maximum likelihood method [27] based on accurate numerical calculations of the stable densities.

To assess whether the stable model is good, two diagnostics, which were proposed and tested by Nolan [27], were used. Firstly, the smoothed probability density (smoothing was done with a Gaussian kernel [26, 27]) for the data was compared with the fitted stable probability density. Secondly, the variance-stabilized PP (percent-percent) plot of the data versus the fit was compared with a 45-degree straight line. The stable model is good if there is good agreement between the fitted stable density and the smoothed data density, and the PP plot is essentially on the 45-degree line [27]. In the PP (percent-percent) plot, the value of the empirical cumulative distribution function (CDF) at each data point is plotted against the value of the fitted stable CDF at the corresponding point. If the stable fit is good, the two values (in percentages) for each plotted point would be close and thus the plot would lie close to the 45-degree line. The fitted stable parameters reported here are in the S0 parameterization [26], corresponding to the respective parameters in the characteristic function given above.

## Supporting Information

S1 Table Fitted *β* and *δ* parameters for experimental IDI fluctuations. (PDF)

S2 Table Fitted *β* and *δ* parameters for simulated IDI fluctuations. (PDF)

S1 Dataset Experimental IDI fluctuations (Threshold -1.5). Filename is the experimental pump parameter. (ZIP)

S2 Dataset Experimental IDI fluctuations (Threshold -2.0). Filename is the experimental pump parameter. (ZIP)

S3 Dataset Simulated IDI fluctuations (Threshold -1.0). Filename is the simulation pump parameter. (ZIP)

S4 Dataset Simulated IDI fluctuations (Threshold -1.5). Filename is the simulation pump parameter. (ZIP)
