# Supplementary material for: Heavy-Tailed Fluctuations in the Spiking Output Intensity of Semiconductor Lasers with Optical Feedback
Source: PLoS One. 2016 Feb 22;11(2):e0150027. doi: 10.1371/journal.pone.0150027 (PMC4767187; doi:10.1371/journal.pone.0150027)
Supplement: S1 Table — (PDF) [file pone.0150027.s001.pdf]

	Threshold -1.5		Threshold -2.0	
Experimental pump parameter, p	beta	delta	beta	delta
0.957	-0.063	2.30E-05	-0.106	2.26E-03
0.964	0.309	4.46E-06	-0.134	4.41E-06
0.971	0.010	3.02E-06	-0.059	3.16E-06
0.978	0.024	4.75E-03	0.626	1.10E-03
0.986	0.274	-1.01E-02	0.224	-8.92E-03
0.993	0.196	-6.97E-03	0.156	-6.91E-03
1.000	0.153	-5.09E-03	0.096	-6.45E-03
1.007	0.177	-5.28E-03	0.201	-1.02E-02
1.014	0.176	-4.87E-03	0.036	-1.77E-06

Stable fitted parameters, beta and delta, for the experimental IDI fluctuations for two intensity thresholds.
