# Supplementary material for: Heavy-Tailed Fluctuations in the Spiking Output Intensity of Semiconductor Lasers with Optical Feedback
Source: PLoS One. 2016 Feb 22;11(2):e0150027. doi: 10.1371/journal.pone.0150027 (PMC4767187; doi:10.1371/journal.pone.0150027)
Supplement: S2 Table — (PDF) [file pone.0150027.s002.pdf]

	Threshold -1.0		Threshold -1.5	
Simulation pump parameter, μ	beta	delta	beta	delta
0.985	0.939	-2.65E-03	0.039	-2.20E-04
0.990	0.030	-4.18E-03	0.703	-2.23E-03
0.995	0.877	-3.16E-02	0.283	-2.74E-04
1.000	0.983	-3.31E-02	0.987	-3.10E-03
1.005	0.932	-3.74E-02	0.990	-6.88E-03
1.010	0.687	-3.27E-02	0.787	-1.64E-02
1.015	0.450	-2.44E-02	0.154	5.04E-03
1.020	0.409	-2.28E-02	0.197	5.95E-05

Stable fitted parameters, beta and delta, for the simulated IDI fluctuations for two intensity thresholds.
